# Single crystal toroidal diamond anvils for high pressure experiments beyond 5 megabar

**DOI:** 10.1038/s41467-018-06071-x

**Published:** 2018-09-03

**Authors:** Zs. Jenei, E. F. O’Bannon, S. T. Weir, H. Cynn, M. J. Lipp, W. J. Evans

**Affiliations:** Physics Division, Physical & Life Sciences Directorate, Lawrence Livermore National Laboratory, Livermore, CA 94551 USA

## Abstract

Static compression experiments over 4 Mbar are rare, yet critical for developing accurate fundamental physics and chemistry models, relevant to a range of topics including modeling planetary interiors. Here we show that focused ion beam crafted toroidal single-crystal diamond anvils with ~9.0 μm culets are capable of producing pressures over 5 Mbar. The toroidal surface prevents gasket outflow and provides a means to stabilize the central culet. We have reached a maximum pressure of ~6.15 Mbar using Re as in situ pressure marker, a pressure regime typically accessed only by double-stage diamond anvils and dynamic compression platforms. Optimizing single-crystal diamond anvil design is key for extending the pressure range over which studies can be performed in the diamond anvil cell.

## Abstract (web summary)

Static pressures exceeding 4 million atmospheres are extremely challenging to achieve, but are necessary for the study of matter that exists under these conditions in natural environments. Here, diamonds anvils with a toroidal design are demonstrated to sustain over 6 million atmospheres in a diamond anvil cell.

## Introduction

The diamond anvil cell (DAC) has been around for over 50 years and has been the primary tool for routinely studying materials up to pressures of ~3 Mbar^1–4^. Experiments over 4 Mbar with in situ pressure determination have been reported, but these reports are both scarce and sporadic^5,6^. This indicates that these experiments are challenging, and that the success rate of these experiments is quite low. These studies used beveled or double-beveled single-crystal diamond anvils with ~20 μm culets, and it seems that further decreasing the culet size does not result in routinely higher pressures (since these reports are seemingly absent in the literature). Recently, pressures as high as 1 TPa have been achieved using a double-stage diamond anvil setup^7^. The approach is to use single-crystal diamonds as the primary anvils and nanocrystalline diamond (NCD) microballs as the secondary anvils (for schematic diagrams of this design see refs.^8,9^). The only other technique that routinely generates pressures over 4 Mbar is dynamic compression experiments^10,11^. However, high temperatures are generated in these types of experiments, and the timescale over which the sample is under high-pressure conditions is extremely short. Another recent study using chemical vapor deposition grown NCD anvils reports pressure achieved in the range of 416–521 GPa at a micron scale location on the irregular surface of the NCD anvils^12^.

Single-crystal diamond is well characterized, and although it is the hardest known material, properties such as hardness and brittleness are anisotropic^13–15^. Cleaving along {110} planes with orientations parallel to the [001] compressive stress may act as the principal trigger for anvil failure^15^. Thus, preventing cleaving along the {110} planes is key in generating ultrahigh pressures. It is known that NCD and/or nanopolycrystalline diamonds (NPD) have high toughness and isotropic mechanical properties due to the randomly oriented nanosized diamond grains^16^. The use of NCD or NPD diamond anvils has been shown to generate pressures up to 1 TPa^7–9^. Interestingly, flat NPD diamond anvils with culets ranging in size from 300 to 500 μm have been shown to generate pressures that are ~1.5–2.0 times higher than their single-crystal anvil counterparts. However, beveled NPD diamond anvils with smaller culets (<200 μm) did not show any significant improvement in maximum pressure generation over single-crystal diamond anvils^16^. Micro-NPD beveled anvils with a culet of 3 µm and a bevel diameter of 10 µm crafted with focused ion beam (FIB) achieve pressures greater than 600 GPa^17^ when measured by the equation of state (EOS) reported in ref.^8^.

One disadvantage to NCD or NPD diamond anvils or double-stage diamond anvils equipped with NCD microballs is that visual and optical studies in the infrared and visible portions of the spectrum are limited. Hence, optimizing single-crystal diamond anvil design is key for extending the pressure range over which optical studies could be performed in the diamond anvil cells (DACs). We do note that there are other challenges associated with optical studies in DACs at multimegabar conditions (e.g., refs.^18,19,20^). Recently, Bassett and Skalwold^15^ proposed that a 27^o^ tilt of the [001] axis with respect to the linear stress axis by rotation around the [100] or [010] may provide the greatest resistance to failure of diamond anvils by cleavage. This intriguing idea presents us with an additional parameter that could be modified to optimize pressure generation with single-crystal diamond anvils. While the proposed change in diamond orientation is intriguing, it also would be very difficult to implement. Ultimately, the maximum achievable pressure of a diamond anvil is related to the yield strength of the anvil. The yield strength of single-crystal diamond has been reported to range from 130 to 200 GPa^20–22^ depending on orientation, and that the maximum achievable pressure is 2.5–3 times higher than the yield strength^21^. Hence, pressures of 600 GPa are consistent with the higher end of reported yield strengths for diamond.

Another issue with generating ultrahigh pressures with single-crystal diamond anvils is that once the culet size is significantly decreased to <20 μm, the gasket material essentially flows away which leads to sample containment issues. Here we utilize a toroidal diamond anvil design first reported by Deweale et al.^23–25^ and Loubeyre et al.^26^. The toroidal anvil is well established in the large volume press community^27^. The toroidal design serves two purposes: (1) it sharply reduces the extrusion of the central portion of the gasket and (2) it decreases the magnitude of the shear stresses in the anvils themselves. Hence, by preventing gasket outflow we can decrease the culet size below 20 μm. This technique allows routine pressure generation over ~4.5 Mbar with experiments as high as ~6.15 Mbar. With further optimization of the toroidal design, and possible implementation of the 27^o^ tilt proposed by Bassett and Skalwold^15^, we fully expect to be within a pressure range that has only been accessible with double-stage DACs or shock wave experiments.

## Results and discussion

### High-pressure experiments with toroidal diamond anvils

**Fig. 1:**
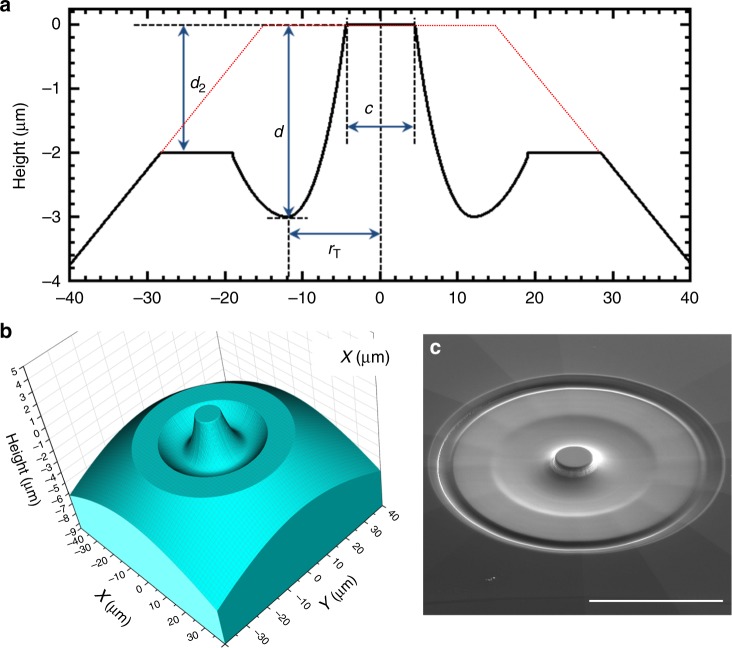
Focused ion beam (FIB) crafted diamond anvil. **a** Cross-section of a FIB modified diamond anvil showing the tuning parameters: culet (*c*), torus radius (*r*_T_), depth of torus (*d*), and depth of outer shoulder *(d*_2_*)*; the red dotted line is the outline of the starting diamond anvil shape. **b** 3D model of a toroidal anvil. **c** Scanning electron microscope image of the modified diamond anvil; scale bar length is 30 µm. Please note the vertical axes in **a**, **b**

Our design is simply a modification of a conventional single beveled diamond anvil. We used FIB to mill a toroidal surface onto the central culet. This configuration achieved pressures well in excess of the highest pressures attained by conventional beveled and double-beveled single-crystal diamond anvils. Moreover, experiments are prepared in a similar manner to a conventional DAC experiment, pre-indenting a Re gasket to a thickness of 5–10 µm, drilling a 3–4 µm diameter hole with a FIB in the gasket, and mounting our sample in the hole (see description in the Methods section). The anvil surfaces were crafted at Lawrence Livermore National Laboratory (LLNL) based on our own designs that underwent several iterations during the past couple of years. Obviously, there are large number of parameters one can tweak to arrive at a configuration suitable to reach 5 Mbar. However, due to the limited experimental time and resources, we focused on tuning the most basic parameters of a toroidal diamond anvil surface: the diameter of the culet, the radius of the torus, and the height of the center disk relative to the culet—this we translated into the maximum depth of the torus—and lastly the height of the flat shoulder outside the torus. These parameters can be seen in Fig. 1a, labeled as *c*, *d*, *r*_T_, and *d*_2_ respectively. The three-dimensional (3D) model only shows the 80 × 80 µm^2^ center culet area of the diamond (Fig. 1b). One of the successful image of the designs taken with a scanning electron microscope (SEM) right after milling is shown in Fig. 1c; on the SEM image one can observe that at the edge of the milled section there is a smoothed step-like feature. This is an artifact of the FIB milling process, and we mitigate this feature by milling in a slope feature over a distance of 4 µm to remove the very sharp edge that is left behind after the initial milling process. Leaving the edge feature will result in a premature failure of the diamonds which is initiated at these sharp edges as they cut through the gasket and become stress concentration points. High-pressure compression experiments for torus parameter optimization were carried out with rhenium gaskets and rhenium as samples, with one single exception when we used a W_25_-Re_75_ alloy gasket.

**Fig. 2:**
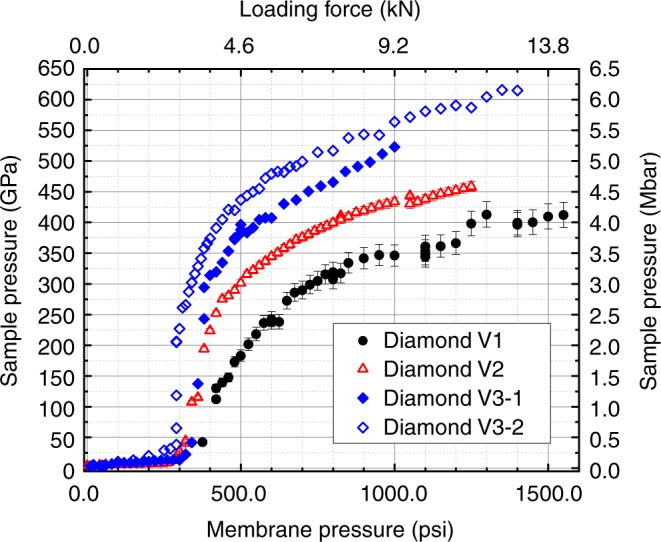
Sample pressure as a function of load of three representative FIB milled toroidal diamond anvil designs. Sample pressure shown as a function of He gas pressure in the membrane of the DAC (bottom axis) and loading force on the diamonds (top axis). Full black circles show pressure from an anvil pair of V1 design, open red triangles are V2 design, and blue diamonds represent pressure points from two pairs of the V3 design anvils, solid blue diamonds first pair and open blue diamonds second pair. Error bars represent experimental uncertainty due to standard deviation in measured atomic volume of the Re

The highest pressures were achieved by diamonds with culets between 8 and 10 µm in diameter, *d* = 3 µm and *d*_2_ = 2 µm, the *r*_T_ parameter values were between 8 and 12 µm. We have experimented with several different designs, choosing parameter values leading to improved performance of the design, i.e., attaining higher pressures. Representative pressure curves as a function of membrane pressure are shown in Fig. 2. The pressure values plotted in this figure, and throughout this paper referring to pressures obtained in present study, have been determined by using fourth-order Birch–Murnaghan EOS with parameters for Re defined in ref.^8^. In order to simplify comparison of our results with those of the double-stage DAC of Dubrovinsky et al.^8^, we have chosen to use the same pressure scale. This pressure scale has been calibrated to 640 GPa and under similar stress–strain environment as the experiments described here. More detailed description of our reasoning for choice of EOS can be found in the Supplementary Discussion. Additional compressions of Pt and Cu together with Re are presented in Supplementary Fig. 1 and Supplementary Fig. 2. Comparison of the maximum attained pressures as determined by using several different EOSs are presented in Supplementary Table 1 and measured lattice parameters, during compression, in Supplementary Tables 2 and Supplementary Table 3, respectively.

**Fig. 3:**
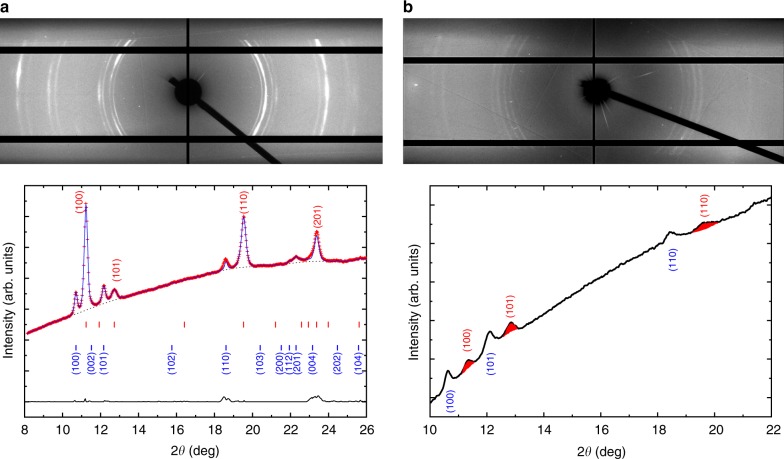
X-ray diffraction patterns above 5 megabar. The top images (both **a**, **b**) are the 2D diffraction patterns showing doubling of the diffraction patterns originating from the low pressure off culet (toroidal well) and high pressure on culet areas. On the bottom panel the integrated diffraction patterns are shown. **a** First pair of V3 diamonds, X-ray wavelength for this pattern *λ* = 0.405473 Å, the dotted line represents the background subtracted before Rietveld refinement, blue line is the refined pattern, and the black continuous line is the residual to the experimental data (red+). Red ticks represent the high-pressure Re diffraction peak locations and blue ticks the low-pressure peaks from the toroidal well. **b** Third pair of V3 diamonds, X-ray wavelength *λ* = 0.406626 Å, the shaded diffraction peaks originate from Re on the culet, and blue labeled ones are from the torus, lower pressure

The first versions of the diamonds that exceeded 400 GPa had ~10 µm culets, *r*_T_ parameter values around 8 µm, and a *d*_2_ parameter value of 0.75*d, and are labeled as V1 in Fig. 2. In subsequent versions we adjusted *d*_2_ to 0.66*d value which are labeled V2 and we increased the *r*_T_ parameter to 12 µm in V3. In all these cases the *d* parameter has been kept at 3 µm. The highest pressure achieved by the three variants are 425 (15) GPa, 460 (8) GPa, and  615 (22) GPa, respectively. We have had several successful compressions with V2 design of the diamonds, all following the same general pressure vs load trend shown in red open triangles in Fig. 2, and all of them achieved at least 450 GPa before failure. The solid blue diamonds in Fig. 2. represent the first compression experiment of V3 diamonds. In this experiment we reached 521 (12) GPa, this being the first of three pairs of V3 diamonds we experimented with so far. The third pair of V3 diamonds in similar compression experiment reached 542 (15) GPa. The highest pressure diffraction patterns of the first three pairs of V3 diamonds are shown in Fig. 3. In all of the compression experiments using diamond designs V2 and V3 upon increasing the membrane pressure, the sample shows a relatively slow increase in pressure up to ~300 psi, and above this membrane pressure, the pressure in the center culet area increases very rapidly to beyond 200 GPa. Above ~200 GPa, sample pressure increase continues at a much slower rate.

**Fig. 4:**
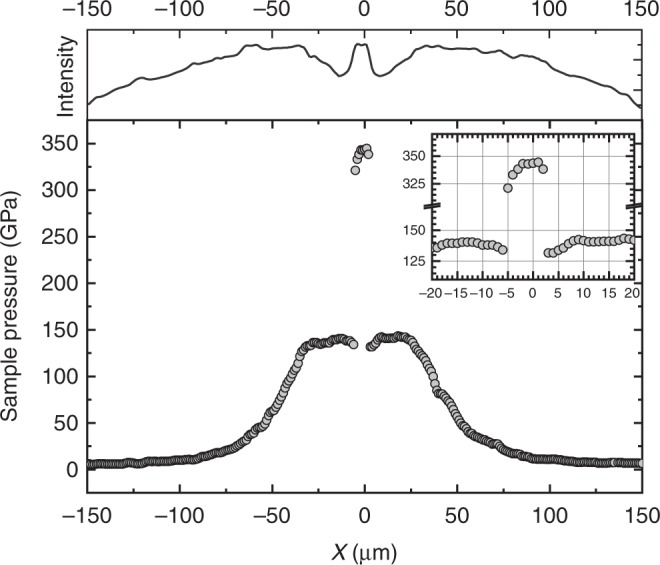
Pressure along the cross-section through the center of a toroidal anvil cell under 580 psi (5.3 kN) load. Diffraction patterns were collected at every 1 µm, and pressure was determined from the measured Re lattice parameters/volume using the EOS from ref. ^8^. The inset shows in more detail the pressure distribution around the culet. Top panel is the corresponding X-ray transmission curve collected with the beamstop diode

The highest pressure diffraction patterns obtained by compressing the first and third pairs of V3 design diamonds are shown in Fig. 3. On the top panel (both 3a, b) a diffraction pattern is shown as collected. It is evident from both patterns that two distinct hexagonal diffraction patterns are present, one that has its origin in the center of the culet—higher pressure Re—and a lower pressure pattern that originates from the torus section of the diamond anvil. This doubling of the diffraction peaks is present in every one of the high-pressure experiments with these small culets, regardless of the gasket material (Re or W_0.25_-Re_0.75_). We note that after the initial pressure jump of around 300 psi membrane pressure, 2.75 kN, each set is following a different compression curve. The doubling of the diffraction peaks is due to the size of the tails of the focused X-ray beam. As described in the experimental section, the full width at half maximum (FWHM) of the X-ray beam is 1.2 × 2.3 µm; however, there is a considerable tail associated with the peak and, additionally, the off-culet section of the Re gasket is considerably thicker than the Re on the central culet. In the lower panel of Fig. 3a, the Rietveld refinement of the integrated diffraction pattern is shown from the first pair of V3-type diamond anvils. We obtained a good refinement (*R* = 0.93%), the lower pressure Re pattern (17% volume fraction of the total intensity) at 246 (5) GPa pressure and the higher pressure pattern at 521 (12) GPa (83%). In Fig. 3b in the lower panel, we plot the integrated diffraction intensity from the third pair of the V3 diamonds when at the highest pressure. As it can be seen, the peak intensities are quite a bit smaller compared to the other pattern, and the accessible *d*-space is more limited as well, having only three diffraction peaks present in the pattern (100, 101, and 110). The refinement was difficult with so few and low-intensity peaks. Instead, we fitted the individual diffraction peaks, and used them to determine the cell parameters and unit cell volume of the Re at the center of the culet, labeled in red on the figure. We perform least square fitting on the 100, 101, and 110 diffraction peaks (for detailed description see Supplementary Note) and we obtain 542 (15) GPa for the culet pressure. The peaks labeled with blue represent the lower pressure Re diffraction lines originating from the torus section of the diamond anvil, and these peaks show Re at a pressure of 191 (4) GPa. This is a very large gradient from the center culet, but it is similar to the gradient shown in Fig. 4, bottom panel. A complete cross-sectional pressure diagram is shown in bottom panel of Fig. 4. It was collected at a membrane pressure of 580 psi, equivalent to 5.3 kN loading on the anvils, and pressure on the culet was ~344 GPa. We scanned the entire 300 µm beveled section by collecting both X-ray transmission values from the beamstop diode and X-ray diffraction patterns every 1 µm. The pressure was determined using the same EOS as for the sample area. Across the diamond tip the pressure increases from ambient, at the edges (150 µm from the center) to the highest in the center of the culet area. It shows a pressure gradient larger than 200 GPa from the sample to the torus, in just a 4–5 µm radius difference. On the top section of Fig. 4 the X-ray transmission intensity is plotted as a function of the position relative to the culet center. According to this, the thinnest area of the sample is—as expected—at the culet of the anvil, and there is a more absorbing thicker section in the torus. Also interesting is that in the X-ray transmission profile of our diamond anvils, even at near 350 GPa, we do not observe cupping of the anvils—as is usually the case with beveled anvils above pressures of 100–200 GPa. It is also worth noting that the pressure in the milled area of the diamond, except for the culet and the adjacent 3–5 µm, is nearly constant at 138–141 GPa. The area within 3–5 µm from the edge of the culet seems to be at a slightly lower pressure (see Fig. 4) compared to the outer area of the torus. The largest pressure gradient, however, is observed between the culet and the rest of the milled area, and in this case, it is slightly above 200 GPa, a pressure difference that increases as the sample pressure reaches higher values, and it is typical for all our experiments with toroidal anvils.

In this study we demonstrate the feasibility of using FIB milled toroidal diamond anvils to drastically extend the pressure range of single-crystal diamond anvils. Based on these initial experiments, diamond designs V2 and V3 appear to be the best candidates for pressure generation of ~450 and 550 GPa, respectively. The V3 version of the diamonds so far show a remarkable success rate of three out of five reaching pressures above 5 megabars. With this toroidal anvil design, we can use a traditional gasket and sample arrangement, and we show that there is only small pressure gradient in the center 4–6 µm diameter section of the flat culet. A small 3–4 µm sample chamber can be milled out with FIB techniques from the pre-indented gasket and this can be filled completely with a sample and pressure marker. Moreover, as this design uses the same mechanism for sample loading and initial sample containment as a traditional DAC, it could even be loaded with soft pressure media such as He or Ne. This new toroidal diamond anvil design has proven to reach pressures well beyond the limits of a traditional single-crystal DAC experiment. This technique offers the potential to study molecular solids, such as H_2_, N_2_, and H_2_O, and other planetary ices to pressures more than 500 GPa with X-ray and optical techniques. Moreover, additional tuning of the torus parameters, bevel diameter/angle, and different anvil orientations can still be explored. We are confident that pressures more than 650 GPa are within reach of this toroidal diamond anvil technique.

## Methods

### Fabrication of diamond anvils

Diamond anvils with toroidal surfaces were fabricated using standard anvil design beveled diamond anvils by milling away from the beveled surface to form the designed shape. The starting anvils were standard Type Ia 16 faceted diamonds, selected for low fluorescence and low birefringence. Beveled culets of 30–40 µm on 300 µm with an 8.5^o^ degree bevel were used depending on the design parameters. The toroidal surfaces of the diamonds are generated by our own codes (Fig. 1b) and then translated into a bitmap (Supplementary Fig. 3) that can be used in the FIB instrument to mill the toroidal surface onto the flat surface of the beveled diamond anvil. The milling is done using 9 nA or 13 nA of 30 keV Ga ions. During the process of milling implantation of the Ga ions into the top 25–30 nm of the diamond surface creates some damage in the crystalline diamonds. We have experimented with removing this layer by oxygen plasma etching. Transmission electron microscopy results show that the damaged layer has been removed, but we have not noticed any significant performance (i.e., highest pressures reached) difference between the plasma etched and non-etched diamonds. Thus, the results that we are presenting in this paper all used FIB milled diamonds that were not plasma etched after milling.

### DAC experiments

LLNL-designed membrane diamond anvil cells (mDACs)^28^ were used for all high-pressure experiments. Diamond anvils for different experimental runs were described above. We used 0.25 mm thick Re disks as gasket starting material. To obtain an indented thickness of less than 10 μm we employed a two-step indentation: first the Re gaskets were pre-indented to a thickness of 20–25 μm using a pair of 50/300 μm single beveled diamonds. Then, these gaskets were mounted into the mDAC equipped with the toroidal diamond anvils and indented to a thickness of ~4–5 μm. The sample chamber is formed by drilling a 3–4 µm hole with FIB in the center of the 9 µm indented section. Re served as both the pressure marker and sample in all experiments, and we used the fourth-order EOS of Re reported by Dubrovinsky et al.^8^.

### X-ray diffraction measurements

Angle-dispersive X-ray diffraction experiments were carried out at the High-Pressure Collaboration Access Team (HPCAT) beamline 16ID-B at the Advanced Photon Source at Argonne National Laboratory, at Sector 16. Diffraction patterns were collected at room temperature over the course of several different experimental runs using monochromatic X-ray beams typically 30 keV. The diffraction patterns were collected with a Pilatus 1M-F with typical exposure times between 4 and 8 s. The beam was focused using the 200 × 100 mm KB mirror assembly which results in a beam size FWHM of ~1 × 2 μm^2^—as measured using a knife edge assembly—with a much lower intensity tail extending beyond this region. The two-dimensional (2D) images collected were integrated using DIOPTAS^29^ to obtain an intensity curve of the diffraction peaks as a function of the 2θ angle, which were analyzed to obtain the cell parameters using OriginPro software package. Refinement was done with JADE (copyrighted commercial software) version 9, and for profile shape function we used Pseudo-Voigt function for both Re, for preferred orientation vector (100) was optimized for both Re at high pressure and low pressure.

## Electronic supplementary material

Supplementary Information

## Data Availability

All relevant data will be made available upon reasonable request from the corresponding author.
