# Supplementary material for: Single crystal toroidal diamond anvils for high pressure experiments beyond 5 megabar
Source: Nat Commun. 2018 Sep 3;9:3563. doi: 10.1038/s41467-018-06071-x (PMC6120914; doi:10.1038/s41467-018-06071-x)
Supplement: Supplementary file 1 — Supplementary Information [file 41467_2018_6071_MOESM1_ESM.pdf]

SUPPLEMENTARY INFORMATION

Single crystal toroidal diamond anvils for high pressure experiments beyond 5 megabar

Zs. Jenei et. al

Supplementary Discussion

Having the most accurate EOS to determine the pressure of the sample is very important in high pressure experiments. Therefore, we chose an equation of state that has been calibrated against an EOS of Au derived from shock compression to 550 GPa. Moreover, the stress-strain conditions of our samples are more closely related to the conditions under which authors in ref. 1 have made their calibration. We recognize that this is not the latest EOS, however, the latest publication on Re EOS (Anzellini et al)[1] calibrated the fitting parameters up to 144 GPa. Sakai et al.[2] however, in the conclusion state, and we agree, that extrapolating EOSs to more than four times the calibration pressure is far beyond what is rationalized by experimental data. Additionally, we have collected x-ray diffraction data of Re and Pt and Re and Cu when compressed together (thin layer of Pt/thick Cu on Re at the center of the culet), up to 300 GPa (Supplementary Figure 1, and Supplementary Figure 2). As the two figures illustrate, up to 300 GPa there is good agreement between the used Re pressure scale versus the Pt scale, after Yokoo et al.[3] In case of Cu, there is a good agreement below 70 GPa and above 240 GPa, whereas in the intermediate pressures a large discrepancy in the pressure values is observed. It should be noted that the 4th order Birch-Murnaghan EOS does not yield us the highest pressure values (see Supplementary Table 1), however, based on what is available in the literature, we consider it to be the most suitable EOS for our experiments.

For comparison we listed the maximum pressures attained by three of our V3 diamonds with a list of equations of state in supplementary Table 1, see below. For completeness we also list our obtained cell parameter values for the two of the highest pressure runs in Supplementary Table 2, and Supplementary Table 3.

Supplementary Note

Cell parameters in Supplementary Table 2 and Supplementary Table 3 were calculated as follows: We fit the individual 100, 101 and 110 diffraction peaks and obtained the *d*-spacings for these peaks and the standard deviation from the fitting. Then we calculate a_{100} and a_{110} parameter (and the standard deviation) of the hexagonal lattice from the d_{100} and d_{110}. Then we use the two a parameters and the d_{101} to calculate $c_{100\&101}$ and $c_{110\&101}$ parameters and standard deviation. The last step is to perform a least square procedure to obtain the a and c parameters and associated errors. These values are listed in the Supplementary Table 2 and Supplementary Table 3.

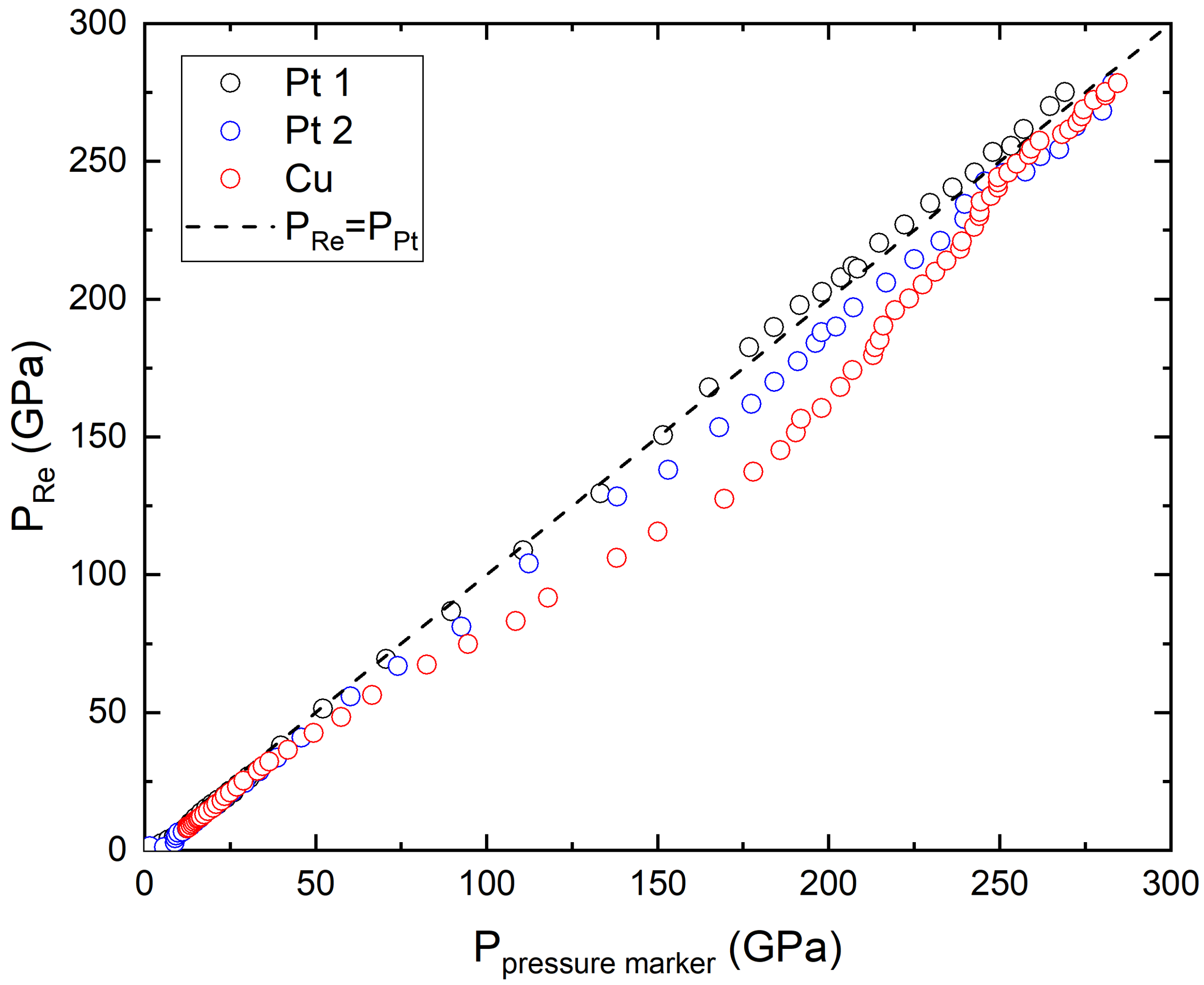

Supplementary Figure 1. Pressure of the central culet area determined by the Re scale reported by Dubrovinsky et al.[4]as a function of the pressure determined by the Pt scale reported by Yokoo et al.[3] and Cu scale reported by Holzapfel et al[5]. The dashed line corresponds to $P_{Re}=P_{Pt}$. In one Pt experiment the maximum pressure determined by the Pt scale reported by Yokoo et al.[3] is less than 10 GPa higher than the pressure determined by the Re scale reported by Dubrovinsky et al.[4]. In the other Pt experiment the Re scale shows a pressure ~10 GPa higher than the Pt scale. Also note that we compared the Pt scales of Holmes et al.[6] and Yokoo et al.[3]and the difference at the maximum pressure of both the Pt experiments is < 4 GPa. In the Cu experiment the maximum pressure determined by Cu is ~10 GPa higher than the Re pressure.

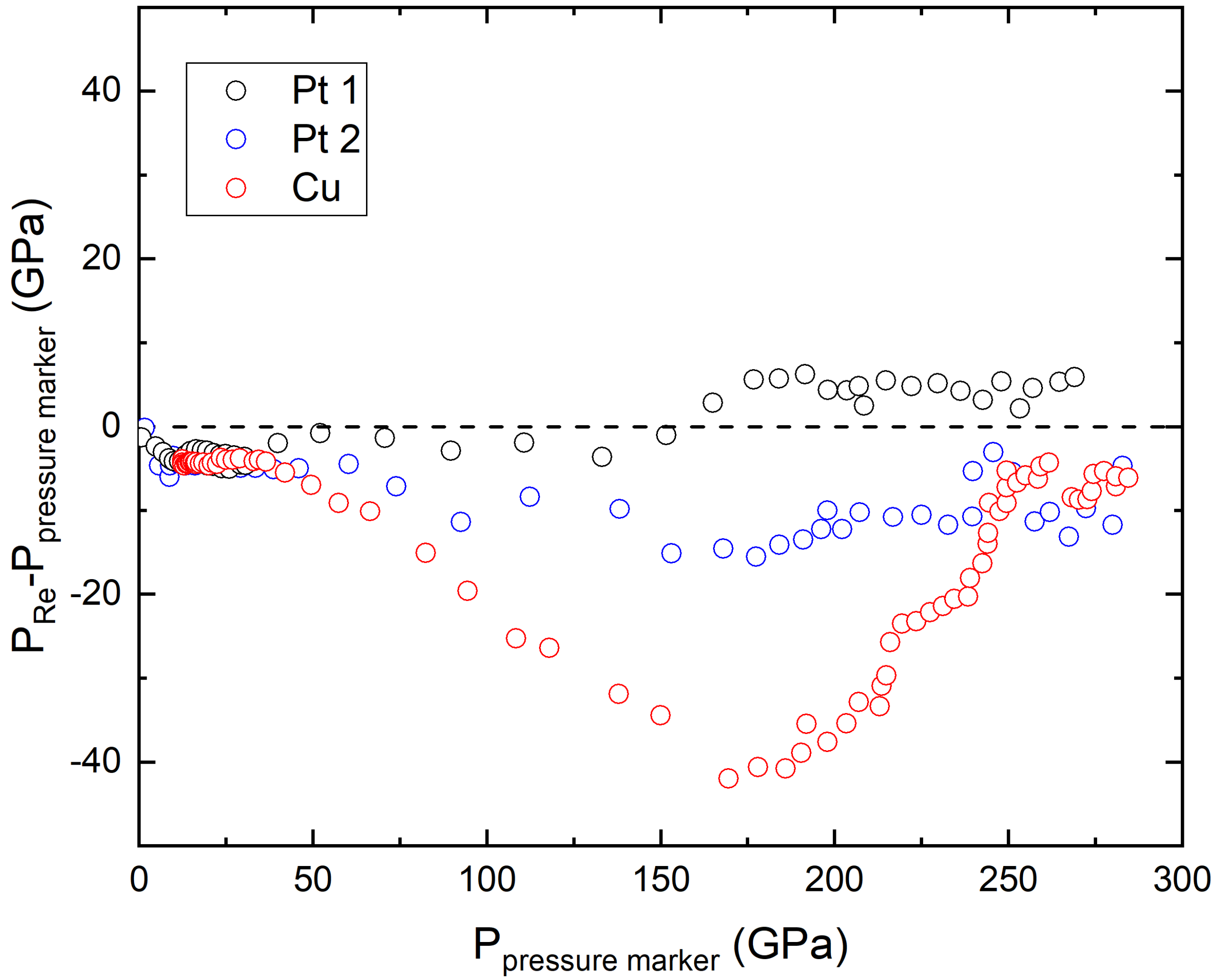

Supplementary Figure 2. P_{Re}-P_{sample} as a function of sample pressure determined by Pt Yokoo et al.[3] . Two of the three experiments show that the pressure determined by the EoS of Re reported by Dubrovinsky et al.[4] underestimates the maximum pressure by as much as ~10 GPa at the maximum pressure of these experiments. We note that in the lower pressure region, the Cu shows a large pressure difference, only to converge to near the Re-pressure values above 240 GPa.

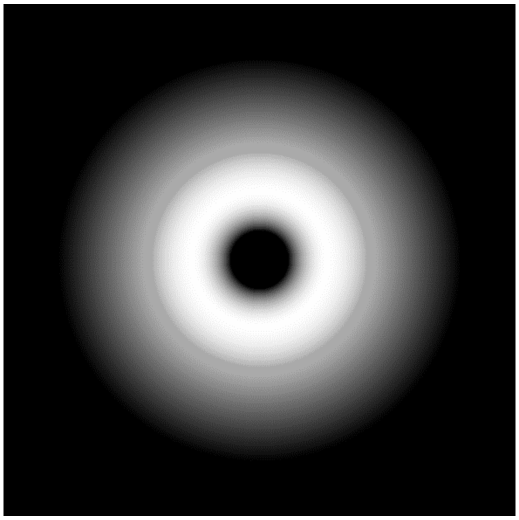

Supplementary Figure 3. Bitmap used to mill a typical toroidal diamond design used in this study.

Supplementary Table 1. The maximum pressure of three different experiments based on different Re EoS's reported in the literature.

Reference	EoS	P-Range (GPa)	K (GPa)	K'	K''	PTM	Pressure Gauge	Method	FD25 Max P (GPa)	FD35 Max P (GPa)	FD39 Max P (GPa)
Dubrovinsky et al (2012)[4]	BM4	0-640	342	6.15	-0.029	Ne/None	Au	XRD DAC	521(12)	542(15)	615(22)
Dubrovinsky et al (2012)[4]	BM3	0-165	353	5.80		Ne	Au	XRD DAC	508(6)	528(11)	596(19)
Dubrovinsky et al (2012)[4]	Vinet	0-165	348	6.07[a]		Ne	Au	XRD DAC	528(10)	549(12)	620(20)
Sikka, S. K., & Vijayakumar, V (1988)[7]	BM3	Room P to V/Vo = 0.72	372	5.41				LMTO	513(7)	529(12)	599(18)
Manghnani et al. (1974)[8]			360.3	5.43				Ultrasonic	489(9)	506(10)	571(17)
Jeanloz et al. (1991)[9]	BM4	0-170	360.3	5.43	-22.5	4:1, none	Ruby	XRD DAC	486(8)	505(11)	568(17)
Lv et al (2012)[10]	Vinet		376	4.58				GGA-PBE	432(6)	447(8)	498(11)
Anzellini et al (2014)[1]	Vinet	0-144	352.6	4.56		He	Ruby, W, He	XRD DAC	403(8)	447(8)	465(10)
McQueen et al (1970)[11]	Vinet	0-280	365.2	4.35				Reduced Shock	398(6)	417(7)	458(9)
Vohra et al. (1987)[12]	BM3	0-216	372	4.05		?	Ruby, Re	XRD DAC	378(5)	390(8)	432(8)
Liu et al. (1970)[13]	BM2	0-35	336	4[b]		NaCl	NaCl	XRD DAC	335(5)	346(6)	384(7)

[a] The published value for K' = 7.57 reported by [1] was modified by [6] to K' = 6.05 because it was incompatible with the P-V data points reported by [1]. Note that using the published value of 7.57 for K' results in pressures over 7 Mbar.

[b] fixed parameter

Supplementary Table 2. Rhenium lattice parameters from experiment FD25 *a*-axis calculated from 100 and 110, *c*-axis calculated from 101, 100 and 101, 110 using a least squares procedure.

***a*-axis (Å)**	***a* error (Å)**	***c*-axis (Å)**	***c* error (Å)**	**Volume (Å^3)**	**Vol error (Å^3)**
2.75072	0.00076	4.45081	0.00117	29.16508	0.01787
2.75124	0.00095	4.44877	0.00068	29.16262	0.02056
2.75131	0.00109	4.44406	0.00060	29.13325	0.02333
2.74921	0.00105	4.43191	0.00065	29.00937	0.02259
2.74716	0.00102	4.41294	0.00063	28.84208	0.02175
2.74648	0.00081	4.39812	0.00065	28.731	0.01744
2.74574	0.00080	4.39427	0.00067	28.69032	0.01735
2.74499	0.00079	4.39337	0.00070	28.66889	0.01703
2.74398	0.00066	4.3915	0.00068	28.63564	0.01446
2.74256	0.00074	4.38995	0.00069	28.59588	0.01599
2.74083	0.00068	4.38756	0.00069	28.54431	0.01492
2.73895	0.00070	4.38355	0.00074	28.47898	0.01527
2.73673	0.00056	4.38242	0.00073	28.42555	0.0125
2.73422	0.00037	4.38682	0.00063	28.40187	0.00872
2.73135	0.00040	4.39384	0.00065	28.38769	0.00926
2.72765	0.00043	4.408	0.00071	28.40211	0.01
2.71238	0.00083	4.37001	0.00085	27.84292	0.01797
2.66689	0.00245	4.33324	0.00098	26.69034	0.04931
2.6156	0.00345	4.5356	0.00295	26.87255	0.07297
2.48646	0.00084	4.1051	0.00182	21.97945	0.01771
2.47042	0.00101	4.04379	0.00161	21.37272	0.01943
2.4623	0.00113	4.03096	0.00162	21.16511	0.02126
2.45853	0.00073	4.02766	0.00170	21.08313	0.0154
2.45424	0.00082	4.01253	0.00180	20.93072	0.01685
2.44866	0.00123	3.99715	0.00137	20.75577	0.02208
2.44355	0.00142	3.98825	0.00162	20.62323	0.02532
2.43801	0.00146	3.97708	0.00153	20.47223	0.0257
2.43587	0.00071	3.9759	0.00156	20.43023	0.01439
2.43415	0.00050	3.98033	0.00179	20.42418	0.0125
2.43137	0.00073	3.9699	0.00173	20.32414	0.01508
2.42845	0.00079	3.96094	0.00137	20.22958	0.01489
2.42506	0.00069	3.96009	0.00168	20.16882	0.01432
2.42172	0.00054	3.96678	0.00158	20.14728	0.01202
2.41635	0.00071	3.95065	0.00161	19.97654	0.01422
2.41138	0.00050	3.94132	0.00156	19.84745	0.01132
2.40689	0.00062	3.92743	0.00150	19.7039	0.01266
2.40281	0.00050	3.9327	0.00137	19.66347	0.01071
2.401	0.00050	3.92391	0.00120	19.59002	0.01006
2.40036	0.00098	3.90996	0.00122	19.50985	0.0171
2.39748	0.00094	3.90108	0.00090	19.41894	0.01581
2.39521	0.00107	3.89657	0.00116	19.35984	0.01822
2.39332	0.00101	3.89413	0.00114	19.31714	0.01719

Supplementary Table 3. Rhenium lattice parameters from experiment FD39 *a*-axis calculated from 100 and 110, *c*-axis calculated from 101, 100 and 101, 110 using a least squares procedure.

***a*-axis (Å)**	***a* error (Å)**	***c*-axis (Å)**	***c* error (Å)**	**Volume (Å^3)**	**Vol error (Å^3)**
2.76158	0.00371	4.40839	0.00102	29.11555	0.02062
2.75522	0.00034	4.44427	0.00084	29.2176	0.01848
2.74681	0.00425	4.48746	0.00099	29.32174	0.01946
2.75091	0.00085	4.44354	0.00084	29.12134	0.01301
2.74819	0.00072	4.44005	0.00076	29.04113	0.01492
2.74553	0.00082	4.43252	0.00065	28.9357	0.01324
2.74394	0.00129	4.42515	0.00063	28.85408	0.01273
2.74167	0.00109	4.41483	0.00066	28.73919	0.01322
2.74015	0.00109	4.4093	0.00074	28.67138	0.01553
2.73855	0.00093	4.41014	0.00081	28.64349	0.01733
2.73702	0.00073	4.40529	0.00065	28.57991	0.01397
2.73563	0.00068	4.40389	0.00072	28.54175	0.01549
2.73347	0.00042	4.4044	0.00075	28.50003	0.01569
2.73211	0.00041	4.40123	0.00073	28.45122	0.01437
2.73029	0.00052	4.3999	0.00082	28.40487	0.01484
2.72884	0.00059	4.39464	0.00069	28.34074	0.01313
2.72711	0.00035	4.39169	0.00062	28.28579	0.01224
2.72494	0.00030	4.38784	0.00060	28.21609	0.01245
2.72247	0.00045	4.38297	0.00069	28.1336	0.01494
2.71942	0.00076	4.37413	0.00058	28.01398	0.01285
2.71772	0.00086	4.37006	0.00060	27.95286	0.01335
2.71516	0.00060	4.36229	0.00067	27.85076	0.01396
2.71289	0.00092	4.35357	0.00076	27.7486	0.01634
2.71071	0.00143	4.3375	0.00104	27.60174	0.02117
2.70795	0.00085	4.32994	0.00118	27.49758	0.02462
2.70268	0.00065	4.32058	0.00150	27.33149	0.03187
2.70006	0.00436	4.28517	0.00253	27.05487	0.04942
2.70361	0.00204	4.31133	0.00147	27.29159	0.02967
2.70283	0.00255	4.30675	0.00226	27.24691	0.04419
2.70545	0.00111	4.37758	0.00200	27.74871	0.04291
2.59151	0.00056	4.12256	0.00203	23.97741	0.01566
2.53587	0.00068	4.13578	0.00195	23.03251	0.0165
2.532	0.00055	4.07468	0.00134	22.623	0.01235
2.54672	0.04566	4.07668	0.00131	22.89801	0.82104
2.53318	0.00018	4.06345	0.00102	22.58176	0.00648
2.52646	0.00031	4.04649	0.00146	22.36832	0.0098
2.52514	0.00412	4.0579	0.00074	22.40794	0.07325
2.5238	0.00326	4.06342	0.00097	22.41464	0.0581
2.5098	0.00326	4.0486	0.00110	22.08578	0.05776
2.49874	0.00132	4.00708	0.00065	21.66707	0.02318
2.49403	0.00229	4.00509	0.00058	21.57471	0.03976
2.48496	0.00145	3.99039	0.00063	21.33955	0.02519
2.47757	0.00174	3.9779	0.00085	21.14649	0.03009
2.47205	0.00172	3.96621	0.00094	20.99041	0.02966

2.46645	0.00226	3.95427	0.00081	20.83248	0.03841
2.46228	0.00166	3.94695	0.00081	20.72363	0.02828
2.45772	0.00118	3.93189	0.00079	20.56814	0.02023
2.45435	0.00108	3.92743	0.00091	20.48866	0.0187
2.45134	0.00093	3.92203	0.00085	20.41033	0.01609
2.4472	0.00099	3.90523	0.00089	20.25428	0.01701
2.44164	0.00117	3.89619	0.00099	20.11564	0.01993
2.43838	0.00113	3.8902	0.00102	20.0312	0.01935
2.43476	0.00288	3.88347	0.00098	19.93709	0.0475
2.43101	0.00089	3.87892	0.00105	19.85247	0.01546
2.42848	0.00264	3.86503	0.00116	19.74019	0.0433
2.4254	0.00287	3.86478	0.00105	19.68892	0.04687
2.42335	0.00403	3.8576	0.00132	19.6191	0.06568
2.41872	0.00066	3.86815	0.00130	19.59767	0.01253
2.41531	0.00056	3.86646	0.00125	19.53392	0.01107
2.41292	0.00090	3.8648	0.00131	19.48688	0.01599
2.41181	0.00288	3.85807	0.00149	19.43515	0.047
2.40927	0.00245	3.85497	0.00154	19.37856	0.04018
2.40765	0.00272	3.85648	0.00140	19.3601	0.04432
2.40577	0.00293	3.84986	0.00154	19.29678	0.04767
2.4018	0.00263	3.84319	0.00140	19.19985	0.04258
2.39932	0.00423	3.8394	0.00172	19.14124	0.068
2.39656	0.00180	3.83306	0.00163	19.06566	0.02971
2.39482	0.00229	3.82858	0.00156	19.01577	0.03726
2.39531	0.00159	3.83326	0.00174	19.04682	0.02671
2.39252	0.00087	3.82344	0.00152	18.9538	0.01567
2.39094	0.00147	3.80922	0.00150	18.85832	0.02443
2.38932	0.00065	3.80842	0.00170	18.82888	0.01321
2.38704	0.00117	3.80624	0.00141	18.78221	0.01968
2.38413	0.00073	3.81248	0.00159	18.76721	0.01389
2.38363	0.00190	3.80907	0.00119	18.74258	0.03042
2.38192	0.00092	3.80681	0.00102	18.70448	0.01526
2.38097	0.00076	3.79601	0.00113	18.63649	0.01309
2.37906	0.00072	3.79868	0.00091	18.61982	0.0121
2.37906	0.00072	3.79868	0.00091	18.61982	0.0121

Supplementary References

1 Anzellini, S., Dewaele, A., Occelli, F., Loubeyre, P. & Mezouar, M. Equation of state of rhenium and application for ultra high pressure calibration. *Journal of Applied Physics* **115**, 6, (2014).

2 Sakai, T. *et al.* High pressure generation using double-stage diamond anvil technique: problems and equations of state of rhenium. *High Pressure Research* **38**, 107-119, (2018).

3 Yokoo, M. *et al.* Ultrahigh-pressure scales for gold and platinum at pressures up to 550 GPa. *Physical Review B* **80**, (2009).

4 Dubrovinsky, L., Dubrovinskaia, N., Prakapenka, V. B. & Abakumov, A. M. Implementation of micro-ball nanodiamond anvils for high-pressure studies above 6 Mbar. *Nature Communications* **3**, 7 (2012).

5 Holzapfel, W. B. & Nicol, M. F. Refined equations of state for Cu, Ag, and Au in the sub-TPa region. *High Pressure Research* **27**, 377-392 (2007).

6 Holmes, N. C., Moriarty, J. A., Gathers, G. R. & Nellis, The equation of state of platinum to 660 GPa (6.6 Mbar). *Journal of Applied Physics* **66**, 2962-2967 (1989).

7 Sikka, S. K. & Vijayakumar, V. Theoretical isothermal equation of state of rhenium. *Physical Review B* **38**, 10926-10928 (1988).

8 Takemura, K. in *Proceedings of AIRAPT-17.* (ed W.J. Nellis M.H. manghnani, M.F. Nicol) 443-444 (University Press).

9 Jeanloz, R., Godwal, B. K. & Meade, C. Static strength and equation of state of rhenium at ultra-high pressures. *Nature* **349**, 687-689, (1991).

10 Lv, M.-B., Cheng, Y., Qi, Y.-Y., Ji, G.-F. & Piao, C.-G. Elastic properties and phonon dispersions of rhenium in hexagonal-close-packed structure under pressure from first principles. *Physica B-Condensed Matter* **407**, 778-783 (2012).

11 McQueen, R. G., Marsh, S. P., Tayler, J. W., Fritz, J. N. & Carter, W. J. *High-Velocity Impact Phenomena*. (Academic, 1970).

12 Vohra, Y. K., Duclos, S. J. & Ruoff, A. L. High Pressure x-ray diffraction studies on rhenium to 216 GPa (2.16 Mbar). *Physical Review B* **36**, 9790-9792 (1987).

13 Liu, L. G., Takahashi, T. & Bassett, W. A. Effect of pressure and temperature on lattice parameters of rhenium. *Journal of Physics and Chemistry of Solids* **31**, 1345 (1970).
